# Dichloridotetra­kis­(1*H*-1,2,4-triazole-κ*N* ^4^)copper(II)

**DOI:** 10.1107/S1600536812008872

**Published:** 2012-03-03

**Authors:** Maja Vidmar, Tatjana Kobal, Bojan Kozlevčar, Primož Šegedin, Amalija Golobič

**Affiliations:** a Faculty of Chemistry and Chemical Technology, University of Ljubljana, Aškerčeva 5, 1000 Ljubljana, Slovenia

## Abstract

The central Cu^II^ atom of the molecular title complex, [CuCl_2_(C_2_H_3_N_3_)_4_], is situated on a site with symmetry 2.22. It is six-coordinated in an elongated octa­hedral geometry, with the equatorial plane defined by four N atoms of four 1,2,4-triazole ligands and the axial positions occupied by two Cl atoms situated on a twofold axis. The mol­ecules are connected *via* N—H⋯Cl hydrogen bonds and the crystal consists of two inter­penetrating three-dimensional hydrogen-bonded frameworks.

## Related literature

For the synthesis and structure of copper(II) coordination compounds with 1,2,4-triazole derivatives, see: Zhang *et al.* (2003 ▶); Zhang & Wu (2005 ▶); Zhao *et al.* (2009 ▶); Haasnoot (2000 ▶). For the synthesis and structure of 1,2,4-triazole with other metal ions, see: Arion *et al.* (2003 ▶), Haasnoot (2000 ▶). For properties of some Cu^II^ complexes of pesticides, see: Kamiya & Kameyama (2001 ▶); Morillo *et al.* (2002 ▶).
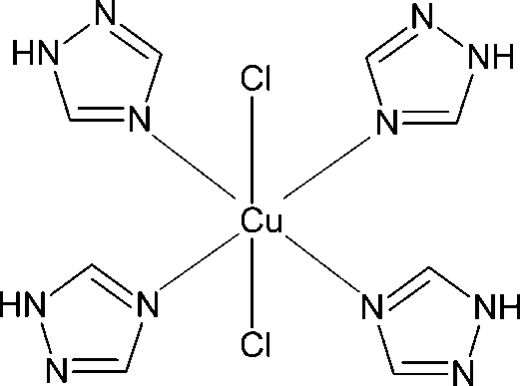


## Experimental

### 

#### Crystal data

[CuCl_2_(C_2_H_3_N_3_)_4_]
*M* *_r_* = 410.75
Tetragonal, 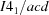

*a* = 14.4471 (3) Å
*c* = 15.8181 (3) Å
*V* = 3301.53 (12) Å^3^
*Z* = 8
Mo *K*α radiation
μ = 1.67 mm^−1^
*T* = 294 K
0.30 × 0.24 × 0.22 mm

#### Data collection

Nonius KappaCCD diffractometer
Absorption correction: multi-scan (*DENZO-SMN*; Otwinowski & Minor, 1997 ▶) *T*_min_ = 0.635, *T*_max_ = 0.711
21092 measured reflections
952 independent reflections
776 reflections with *I* > 2σ(*I*)
*R*_int_ = 0.035

#### Refinement

*R*[*F* ^2^ > 2σ(*F* ^2^)] = 0.025
*wR*(*F* ^2^) = 0.067
*S* = 1.10
952 reflections
59 parameters
H atoms treated by a mixture of independent and constrained refinement
Δρ_max_ = 0.40 e Å^−3^
Δρ_min_ = −0.26 e Å^−3^

### 

Data collection: *COLLECT* (Nonius, 2000 ▶); cell refinement: *DENZO-SMN* (Otwinowski & Minor, 1997 ▶); data reduction: *DENZO-SMN*; program(s) used to solve structure: *SIR08* (Burla *et al.*, 2007 ▶); program(s) used to refine structure: *SHELXL97* (Sheldrick, 2008 ▶); molecular graphics: *ORTEP-3 for Windows* (Farrugia, 1997 ▶) and *Mercury* (Macrae *et al.*, 2008 ▶); software used to prepare material for publication: *WinGX* (Farrugia, 1999 ▶).

## Supplementary Material

Crystal structure: contains datablock(s) I, global. DOI: 10.1107/S1600536812008872/gk2463sup1.cif

Structure factors: contains datablock(s) I. DOI: 10.1107/S1600536812008872/gk2463Isup2.hkl

Additional supplementary materials:  crystallographic information; 3D view; checkCIF report

## Figures and Tables

**Table 1:** Selected bond lengths (Å)

Cu1—N1	2.0049 (12)
Cu1—Cl1	2.8296 (6)

**Table 2:** Hydrogen-bond geometry (Å, °)

*D*—H⋯*A*	*D*—H	H⋯*A*	*D*⋯*A*	*D*—H⋯*A*
N2—H2⋯Cl1^i^	0.80 (2)	2.28 (2)	3.0626 (16)	164 (2)

Symmetry code: (i) 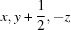.

**Fig. 1.**
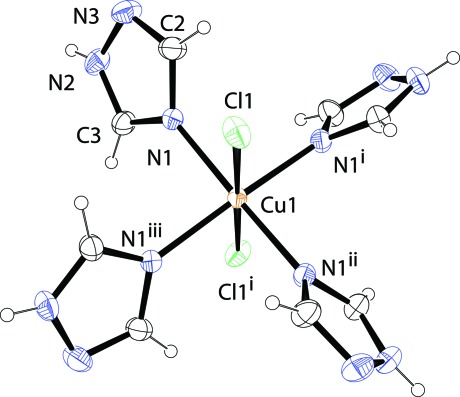
The molecular structure of the title compound showing the atom-labelling scheme. Displacement ellipsoids are drawn at the 30% probability level and H atoms are drawn as small spheres of arbitrary radii. [Symmetry codes: (i) -x, -y + 1/2, z; (ii) -y + 1/4, -x + 1/4, -z + 1/4; (iii) y - 1/4, x + 1/4, -z + 1/4.]

**Fig. 2.**
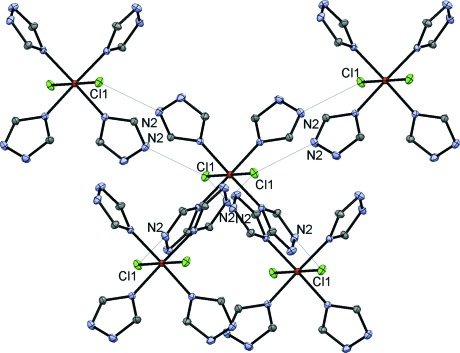
Fragment of a three-dimensional hydrogen-bond framework formed via N—H···Cl intermolecular interactions.

## supplementary crystallographic information

### Comment

The 1,2,4-triazoles are being widely used as pharmaceutical and as agricultural chemicals (Haasnoot, 2000). There has also been considerable research on complexation of pesticides with metal ions since it influences their pharmacological and toxicological properties (Arion *et al.*, 2003; Zhang *et al.*, 2003; Kamiya & Kameyama, 2001; Morillo *et al.*, 2002). We report here the preparation and structure of the novel Cu^II ^complex, (I), containing the 1-*H*-1,2,4-triazole ligands.

The title compound [CuCl_2_(C_2_H_3_N_3_)_4_] is mononuclear complex, where central Cu^II^ atom has a distorted (4 + 2) octahedral coordination environment with four N atoms of 1-*H*-1,2,4-triazole ligands in the equatorial plane and two axial *trans* positioned chlorido ligands. The Cu—N and Cu—Cl bond distances (Table 1) indicate Jahn-Teller elongation of the coordination octahedron. Similar coordination bond lengths and elongation were observed also in all three known structures of analogous mononuclear Cu^II^ complexes containing four coordinated triazolo derivatives and two choride ions at axial position (Zhang & Wu, 2005; Zhang *et al.*, 2003; Zhao *et al.*, 2009). Figure 1 shows the *ORTEP* drawing of complex molecule of (I). The Cu atom lies on a cross-section of three twofold rotation axes (Wyckoff position *b*) and both Cl atoms from the molecule lie on one of these twofold axes (Wyckoff position *f*). Conformation of the molecule is a propeller like. N2 atom is a donor of intermolecular hydrogen bond accepted by Cl atom (symmetry code: *x*, *y* + 1/2, -*z*) from neighbouring molecule. This way molecules are linked into a three-dimensional hydrogen-bonding framework (Figure 2, Table 2). The crystal of (I) consists of two interpenetrating three-dimesional hydrogen-bond frameworks.

### Experimental

To a solution of hydrated copper(II) nitrate(V) (0.196 g, 0.81 mmol) in distilled water (40.0 ml) was added a solution of 37% hydrochloric acid (4.0 ml). The pale blue solution was heated till boiling and the colour changed into green. To a cooled solution was added borax (0.400 g, 1.05 mmol) and 1,2,4-triazole (9.600 g, 0.140 mol). The dark blue solution was obtained and at the end NaCl (7.00 g, 0.120 mol) was added. The solution was left for 48 h and the blue crystals suitable for X-ray analysis were obtained.

### Refinement

H atoms were positioned geometrically and allowed to ride on their parent atoms [C—H = 0.93 Å for aromatic H atoms and *U*_iso_(H) = 1.2 times *U*_eq_(C)] with exception H atom bonded to N atom which was freely refined isotropically.

### Crystal data

**Table:**

[CuCl_2_(C_2_H_3_N_3_)_4_]	*D*_x_ = 1.653 Mg m^−^^3^
*M**_r_* = 410.75	Mo *K*α radiation, λ = 0.71073 Å
Tetragonal, *I*4_1_/*a**c**d*	Cell parameters from 1966 reflections
Hall symbol: -I 4bd 2c	θ = 2.6–27.5°
*a* = 14.4471 (3) Å	µ = 1.67 mm^−^^1^
*c* = 15.8181 (3) Å	*T* = 294 K
*V* = 3301.53 (12) Å^3^	Prism, dark blue
*Z* = 8	0.30 × 0.24 × 0.22 mm
*F*(000) = 1656	

### Data collection

**Table:**

Nonius KappaCCD diffractometer	952 independent reflections
Radiation source: fine-focus sealed tube	776 reflections with *I* > 2σ(*I*)
Graphite monochromator	*R*_int_ = 0.035
φ and ω scans	θ_max_ = 27.5°, θ_min_ = 3.4°
Absorption correction: multi-scan (*DENZO-SMN*; Otwinowski & Minor, 1997)	*h* = −13→13
*T*_min_ = 0.635, *T*_max_ = 0.711	*k* = −18→18
21092 measured reflections	*l* = −20→20

### Refinement

**Table:**

Refinement on *F*^2^	Primary atom site location: structure-invariant direct methods
Least-squares matrix: full	H atoms treated by a mixture of independent and constrained refinement
*R*[*F*^2^ > 2σ(*F*^2^)] = 0.025	*w* = 1/[σ^2^(*F*_o_^2^) + (0.0335*P*)^2^ + 1.7204*P*] where *P* = (*F*_o_^2^ + 2*F*_c_^2^)/3
*wR*(*F*^2^) = 0.067	(Δ/σ)_max_ < 0.001
*S* = 1.10	Δρ_max_ = 0.40 e Å^−^^3^
952 reflections	Δρ_min_ = −0.26 e Å^−^^3^
59 parameters	Extinction correction: *SHELXL97* (Sheldrick, 2008), Fc^*^=kFc[1+0.001xFc^2^λ^3^/sin(2θ)]^-1/4^
0 restraints	Extinction coefficient: 0.0043 (3)

### Special details

**Table:**

Geometry. All s.u.'s (except the s.u. in the dihedral angle between two l.s. planes) are estimated using the full covariance matrix. The cell s.u.'s are taken into account individually in the estimation of s.u.'s in distances, angles and torsion angles; correlations between s.u.'s in cell parameters are only used when they are defined by crystal symmetry. An approximate (isotropic) treatment of cell s.u.'s is used for estimating s.u.'s involving l.s. planes.
Refinement. Refinement of *F*^2^ against ALL reflections. The weighted *R*-factor *wR* and goodness of fit *S* are based on *F*^2^, conventional *R*-factors *R* are based on *F*, with *F* set to zero for negative *F*^2^. The threshold expression of *F*^2^ > 2σ(*F*^2^) is used only for calculating *R*-factors(gt) *etc*. and is not relevant to the choice of reflections for refinement. *R*-factors based on *F*^2^ are statistically about twice as large as those based on *F*, and *R*- factors based on ALL data will be even larger.

### Fractional atomic coordinates and isotropic or equivalent isotropic displacement parameters (Å^2^)

**Table:**

	*x*	*y*	*z*	*U*_iso_*/*U*_eq_	
Cu1	0.0000	0.2500	0.1250	0.03175 (17)	
Cl1	0.13849 (3)	0.11151 (3)	0.1250	0.0453 (2)	
N1	0.07460 (8)	0.31410 (8)	0.03559 (8)	0.0329 (3)	
N3	0.16037 (11)	0.33828 (11)	−0.07999 (9)	0.0488 (4)	
N2	0.13429 (10)	0.41905 (11)	−0.04391 (10)	0.0436 (4)	
C2	0.12290 (12)	0.27712 (13)	−0.02974 (10)	0.0435 (4)	
H2A	0.1288	0.2137	−0.0380	0.052*	
C3	0.08400 (11)	0.40417 (11)	0.02389 (10)	0.0386 (4)	
H3	0.0589	0.4499	0.0583	0.046*	
H2	0.1460 (16)	0.4665 (16)	−0.0679 (13)	0.059 (6)*	

### Atomic displacement parameters (Å^2^)

**Table:**

	*U*^11^	*U*^22^	*U*^33^	*U*^12^	*U*^13^	*U*^23^
Cu1	0.03525 (19)	0.03525 (19)	0.0247 (2)	−0.01284 (14)	0.000	0.000
Cl1	0.0398 (2)	0.0398 (2)	0.0562 (4)	0.0036 (2)	−0.01066 (18)	−0.01066 (18)
N1	0.0358 (7)	0.0325 (6)	0.0305 (6)	−0.0064 (5)	0.0031 (5)	−0.0016 (5)
N3	0.0550 (9)	0.0519 (9)	0.0395 (8)	0.0014 (7)	0.0144 (7)	0.0045 (7)
N2	0.0465 (8)	0.0386 (8)	0.0457 (8)	−0.0059 (6)	0.0072 (7)	0.0109 (7)
C2	0.0573 (11)	0.0370 (8)	0.0362 (8)	−0.0013 (8)	0.0102 (8)	−0.0021 (7)
C3	0.0410 (9)	0.0337 (8)	0.0411 (9)	−0.0021 (7)	0.0060 (7)	0.0019 (7)

### Geometric parameters (Å, º)

**Table:**

Cu1—N1	2.0049 (12)	N3—N2	1.353 (2)
Cu1—Cl1	2.8296 (6)	N2—C3	1.313 (2)
N1—C3	1.321 (2)	N2—H2	0.80 (2)
N1—C2	1.357 (2)	C2—H2A	0.9300
N3—C2	1.306 (2)	C3—H3	0.9300
			
N1^i^—Cu1—N1^ii^	173.87 (7)	Cl1^i^—Cu1—Cl1	180.0
N1^i^—Cu1—N1	90.27 (7)	C3—N1—C2	103.20 (13)
N1^ii^—Cu1—N1	90.06 (7)	C3—N1—Cu1	127.51 (10)
N1^i^—Cu1—N1^iii^	90.06 (7)	C2—N1—Cu1	129.20 (11)
N1^ii^—Cu1—N1^iii^	90.27 (7)	C2—N3—N2	102.21 (13)
N1—Cu1—N1^iii^	173.87 (7)	C3—N2—N3	110.94 (14)
N1^i^—Cu1—Cl1^i^	86.93 (3)	C3—N2—H2	130.1 (16)
N1^ii^—Cu1—Cl1^i^	86.93 (3)	N3—N2—H2	118.6 (15)
N1—Cu1—Cl1^i^	93.07 (3)	N3—C2—N1	114.23 (15)
N1^iii^—Cu1—Cl1^i^	93.07 (3)	N3—C2—H2A	122.9
N1^i^—Cu1—Cl1	93.07 (3)	N1—C2—H2A	122.9
N1^ii^—Cu1—Cl1	93.07 (3)	N2—C3—N1	109.42 (14)
N1—Cu1—Cl1	86.93 (3)	N2—C3—H3	125.3
N1^iii^—Cu1—Cl1	86.93 (3)	N1—C3—H3	125.3
			
N1^i^—Cu1—N1—C3	120.99 (15)	C2—N3—N2—C3	−0.10 (19)
N1^ii^—Cu1—N1—C3	−52.88 (12)	N2—N3—C2—N1	0.2 (2)
Cl1^i^—Cu1—N1—C3	34.05 (13)	C3—N1—C2—N3	−0.22 (19)
Cl1—Cu1—N1—C3	−145.95 (13)	Cu1—N1—C2—N3	176.42 (12)
N1^i^—Cu1—N1—C2	−54.89 (12)	N3—N2—C3—N1	0.0 (2)
N1^ii^—Cu1—N1—C2	131.24 (15)	C2—N1—C3—N2	0.14 (18)
Cl1^i^—Cu1—N1—C2	−141.83 (13)	Cu1—N1—C3—N2	−176.58 (11)
Cl1—Cu1—N1—C2	38.17 (13)		

Symmetry codes: (i) −*x*, −*y*+1/2, *z*; (ii) *y*−1/4, *x*+1/4, −*z*+1/4; (iii) −*y*+1/4, −*x*+1/4, −*z*+1/4.

### Hydrogen-bond geometry (Å, º)

**Table:**

*D*—H···*A*	*D*—H	H···*A*	*D*···*A*	*D*—H···*A*
N2—H2···Cl1^iv^	0.80 (2)	2.28 (2)	3.0626 (16)	164 (2)

Symmetry code: (iv) *x*, *y*+1/2, −*z*.
